# Unraveling the signaling mechanism behind astrocytoma and possible therapeutics strategies: A comprehensive review

**DOI:** 10.1002/cnr2.1889

**Published:** 2023-09-07

**Authors:** Chowdhury Lutfun Nahar Metu, Sunita Kumari Sutihar, Md Sohel, Fatematuz Zohora, Akayed Hasan, Md. Thandu Miah, Tanu Rani Kar, Md. Arju Hossain, Md Habibur Rahman

**Affiliations:** ^1^ Biochemistry and Molecular Biology Bangabandhu Sheikh Mujibur Rahman Science and Technology University Gopalganj Bangladesh; ^2^ Biochemistry and Molecular Biology Mawlana Bhashani Science and Technology University Tangail Bangladesh; ^3^ Department of Biochemistry and Molecular Biology Primeasia University Dhaka Bangladesh; ^4^ Department of Pharmacy, Faculty of Pharmacy University of Dhaka Dhaka Bangladesh; ^5^ Department of Pharmacy Mawlana Bhashani Science and Technology University Tangail Bangladesh; ^6^ Department of Biotechnology and Genetic Engineering Mawlana Bhashani Science and Technology University Tangail Bangladesh; ^7^ Department of Computer Science and Engineering Islamic University Kushtia Bangladesh

**Keywords:** astrocytoma, grade, mutation, natural products, signaling mechanism, treatment

## Abstract

**Background:**

A form of cancer called astrocytoma can develop in the brain or spinal cord and sometimes causes death. A detailed overview of the precise signaling cascade underlying astrocytoma formation has not yet been revealed, although various factors have been investigated. Therefore, our objective was to unravel and summarize our current understanding of molecular genetics and associated signaling pathways with some possible therapeutic strategies for astrocytoma.

**Recent Findings:**

In general, four different forms of astrocytoma have been identified in individuals, including circumscribed, diffuse, anaplastic, and multiforme glioblastoma, according to a recent literature review. All types of astrocytoma have a direct connection with some oncogenic signaling cascade. Common signaling is MAPK cascade, including Ras‐Raf‐ERK, up‐regulated with activating EGFR/AKT/PTEN/mTOR and PDGFR. Recent breakthrough studies found that BRAF mutations, including KIAA1549: BRAF and BRAF V600E are responsible for astrocytoma progression. Additionally, cancer progression is influenced by mutations in some tumor suppressor genes, such as the Tp53/ATRX and MGMT mutant. As synthetic medications must cross the blood–brain barrier (BBB), modulating signal systems such as miRNA is the primary option for treating patients with astrocytoma. However, available surgery, radiation therapy, and experimental therapies such as adjuvant therapy, anti‐angiogenic therapy, and EGFR‐targeting antibody drug are the usual treatment for most types of astrocytoma. Similar to conventional anticancer medications, some phytochemicals slow tumor growth by simultaneously controlling several cellular proteins, including those involved in cell cycle regulation, apoptosis, metastatic spread, tyrosine kinase, growth factor receptor, and antioxidant‐related proteins.

**Conclusion:**

In conclusion, cellular and molecular signaling is directly associated with the development of astrocytoma, and a combination of conventional and alternative therapies can improve the malignancy of cancer patients.

## 1 INTRODUCTION

Astrocytoma is an invasive brain tumor, one of severe cancer with the worst vital prognosis and a slightly higher survival rate by standard treatment. According to the Global Cancer Statistics 2022; GLOBOCAN, a total of 25 050 patients were newly affected, and 18 280 patients have died from only Brain & other nervous system in the United States.^1^ Despite the lack of a definitive link between environmental factors such as smoking, dietary risk factors, cell phone electromagnetic fields, severe head injuries, occupational risk factors, and exposure to pesticides, the only known causes of brain tumors (rare hereditary syndromes, therapeutic radiation, and immune suppression leading to brain lymphomas) account for a small percentage of cases.^2^, ^3^, ^4^, ^5^, ^6^ Whilst the complications of multiple astrocytomas remain unclear, some early astrocytoma difficulties include headaches, seizures, impaired vision, mental state, vision problems, coordination problems, and speech issues for low‐grade astrocytoma. Several molecular genetic events led to the differentiation of Astrocytoma from grades I‐IV. Among the three key events—the deletion of the gene that acts as a tumor suppressor on chromosome 22q, proliferation of the PDGF system, and inactivation of the Tp53 gene, have been associated with the emergence of grade 2 astrocytoma with increased proliferation and decreased apoptosis. At loci 9p, 11p, 13q, and 19q, CDK4 and suppression of the tumor suppressor gene are associated with the appearance of anaplastic astrocytoma. Overexpression of the EGFR gene in GBM (40%), while the tumor suppressor gene on chromosome 10 is inactivated (60%–85%).^7^, ^8^, ^9^ Even though astrocytoma can be challenging, a study is being conducted on the matter and researchers are succeeding. Additionally, several types can affect the brain and CNS; as a result, unique and in‐depth treatment methods as well as the development of biomarkers that shed light on brain cancer.^10^, ^11^ Only a small percentage of astrocytoma individuals have longer survival times; the majority have severe symptoms that worsen due to a combination of other disorders. However, very few factors have been clearly identified with a sex ratio of 1:4; epidemiological studies have found that men experience a higher prevalence than women. Based on these findings, it has been suggested that neurosteroids, particularly the estrogens found in larger amounts in women's brains, are involved in gliomagenesis and play a neuroprotective role in gliomas. Estrogens can attach to nuclear or membrane receptors and activate numerous interrelated signaling cascades.^12^ Although the molecular subtypes currently used at diagnosis do not account for steroid biosynthesis enzymes or receptors, most of the studies presented in Petrelli et al., 2020, brought the connected findings and allowed the conclusion that gliomas and, in particular, astrocytomas, are hormone sensitive tumors. Some teams have focused on the molecular processes in their response pathways to better understand how estrogens, progestogens, and androgens affect gliomagenesis.^13^ As a result, a central conflict in contemporary technology continues to be how to treat astrocytoma. There are many theories about the best therapeutic approach, but none have been sufficiently proven to be correct. The rationale is that the medications for this disease must pass through the CNS and BBB. Phytochemicals can be a ray of hope compared to other potentially effective treatment approaches, since they can function in a multistep procedure that targets many signaling cascades. Specifically, phytochemicals can influence cell growth and survival, metastasis, angiogenesis, apoptosis, and oncogene inactivation in a variety of in vitro and animal models.^14^, ^15^, ^16^, ^17^ Although the intrinsic signaling pathways targeted by phytochemicals in cancer treatment of cancer is still unknown, previous studies have shown that they can suppress both the STAT NF‐B, PIK/AKT, and MAPK signaling pathways and activate some genes that are responsible for tumor suppressor activity in a variety of cancer models.^18^ Furthermore, some commonly used anti‐cancer medications, such as Taxol, resveratrol, vincristine, quercetin, vinblastine, tetrandrine, and arteannuin for multiple malignant tumors, are made from natural substances.^19^ There was a paucity of knowledge on the genetic alterations and signaling processes underlying this disease prior to the last 10 years. However, previous studies have significantly increased our understanding of the genetic abnormalities and related signals that underlie the evolution of Astrocytoma. The objective of the prior study was to evaluate our knowledge of how Astrocytoma progression is affected by mutation biology, associated signaling, and modern treatments.

## 2 METHODOLOGY

This review article has been written based on a systematic search strategy and preferred reporting items, including astrocytoma, grade I‐IV, signaling mechanism, treatment strategies and phytochemical according to MESH terminology^20^ and PRISMA guidelines.^21^ Well‐known search tools, including Google Scholar, PubMed, Science Direct, Web of Science, and Scopus, were used to retrieve the data covered in this paper. Non‐English articles have been kept out of exploration. BioRender non‐professional software and ChemDraw Professional 16.0 were used to generate chemical compound figures and structures, and Graph Pad Prism and MS word were used for figure generation.

## 3 CLASSIFICATION OF ASTROCYTOMA

Astrocytomas are classified according to their grade, based on the abnormal appearance and behavior of the cells under a microscope. Low‐grade astrocytomas, also known as grade I and II, are slow‐growing tumors less aggressive than high‐grade astrocytomas, classified as grade III or IV. Grade III astrocytomas are considered anaplastic astrocytomas, which means that the cells show more abnormal changes and have a higher growth rate than grade II astrocytomas. Grade IV astrocytomas are known as multiforme glioblastoma, the most aggressive and common form of brain cancer.

As WHO mentioned in their updated classification sincefor 2016–2021, oligoastrocytomas are polymorphic tumors that maycan evoke oligodendrogliomas or astrocytic tumors according to their molecular features. Oligodendrogliomas with tumors in the frontal, parietal, and occipital lobes exhibited a higher likelihood of allelic loss than those with tumors in the temporal lobes, according to tumor site. Although 30% to 70% of oligoastrocytomas have LOH 1p and LOH 19q,^8^, ^9^, ^10^, ^11^, ^12^, ^15^ indicating a genetic resemblance to oligodendrogliomas, only 30% of oligoastrocytomas have mutations in the TP53 gene or LOH 17p,^10^, ^16^ indicating a link to astrocytomas. In particular, LOH 1p and LOH 19q are negatively linked to TP53 mutations.^22^

### 3.1 Circumscribed astrocytoma (WHO grade I)

Circumscribed astrocytoma (CA) arises in the central nervous system.^23^ This grade of astrocytoma often affects adolescents and young people and can develop anywhere in the central nervous system, specifically the cerebellum.^24^ According to the grade I of the World Health Organization, it can also happen in the optic nerve and brain stem.^25^, ^26^ The cerebellum (40%), supratentorial regions (35%), optic nerve and hypothalamus (11%), and brainstem (9%) were the tumor sites with the highest frequency.^27^ Although this grade of astrocytoma has some common symptoms, including headache, severe or frequent vomiting, vision problems, and premature puberty, it is considered a benign tumor by the World Health Organization policy for brain and other central nervous system (CNS) tumors.^28^

### 3.2 Signaling mechanism behind circumscribed astrocytoma

The MAPK pathway, responsible for a variety of initiatives in the brain, including memory formation, pain perception, inducing cortical neurogenesis, and growth of the midbrain and cerebellum, corresponds to changes in the molecular origin.^29^ ERK transcription of ERK is essential for growth and their hyperactivation is a critical component in the emergence and spread of cancer. The most significant signaling cascade among the MAPK signal transduction pathways, the Ras/Ras/MAPK (MEK)/ERK pathway, is essential for the survival and growth of tumor cells.^30^

The mitogen‐activated protein kinase (MAPK)/extracellular signal‐regulated kinase pathway leads to astrocytoma.^31^ More specifically, this signaling pathway may be altered in most patients with astrocytoma. For example, the activation of p38 MAPK is believed to be a potential oncogenic factor that promotes brain tumor growth and chemotherapy resistance in glioma cells by promoting tumor invasion and metastasis and being positively associated with tumor grade.^32^ The most probable cause is the tandem duplication of a 2 Mb fragment of 7q, which results in the fusion of two genes and the production of a transforming fusion protein that contains the N‐terminus of KIAA1549 and the kinase domain of BRAF.^33^ Numerous genetic anomalies, most notably gene fusions between KIAA1549 and BRAF, have been shown to cause this dysregulation of the MAPK pathway.^34^

#### 3.2.1 BRAF mutations

Several phosphorylation events and essential signaling elements of the MAPK pathway play an important role in carcinogenesis. Human cancer typically exhibits an alteration of the RAS‐RAF‐MEK‐ERK‐MAPK (RAS‐MAPK) pathway as a result of aberrant receptor tyrosine kinase activation or gain of function mutations, mainly in the RAS or RAF genes.^35^ The MAPK pathway is initiated by activating a transmembrane receptor tyrosine kinase and binding of phosphate molecule to Raf kinase, which in turn activates BRAF, an intracellular serine/threonine kinase. When MEK1/2 is activated, the ERK1/2 transcription complex is also activated, which leads to various paradoxes, such as cell differentiation and senescence.^36^ BRAF fusions are present in about 70% of PAs, but they are only present in 50%–55% of non‐cerebellar PAs and 80% of cerebellar Pas.^37^ The BRAFV600E mutation (8.9% pediatric and 9.75% adult PCA) and KIAA1549‐BRAF fusions (41.1% pediatric and 25.7% adult) are two alterations of the BRAF gene that demonstrate a variable pattern between different age‐groups in case of the majority circumscribed astrocytoma (PCAs).^38^

##### KIAA1549: BRAF

The BRAF protooncogene causes the most prevalent genomic distortion; cancers with duplication of BRAF showed increased mRNA levels of BRAF mRNA and a downstream target, CCND1, compared to tumors without duplication. Both pharmacological inhibition of MEK1 / 2, rapid downstream phosphorylation sites of BRAF, and stable silencing of BRAF through lentiviral transduction of shRNA prevented proliferation and stopped the formation of advanced tumor cells derived from low‐grade gliomas.^39^ In sporadic circumscribed astrocytoma, KIAA1549‐BRAF is a documented hereditary change resulting in the combination of BRAF protein (f‐BRAF) and increased BRAF mobility. Furthermore, in cerebellar NSCs, the f‐BRAF directive creates Ccl2 functions in a manner that is subordinate to ERK and NF‐B.^40^ The prevalence of combinations of KIAA1549‐BRAF was 24 (75%), accompanied by BRAF V600E and histone H3.3 K27M tests to identify links between these subatomic characteristics and clinical features in a companion group of 32 patients with AP. Ten of the 24 patients (or 42%) had the 16–9 combination, eight patients (or 33%) had only the 15–9 combination and one patient (or 4%) had only the 16–11 combination.

##### BRAF V600E

The change in BRAF (V600E) is likely related to the extracerebellar region (*p* = .009) and the progression of diencephalic tumors (4/12; 33%). Missense changes of the V600E type established a large margin in the enormous astrocytoma.^41^ Changes in BRAFV600E in PAs are thought to occur twice. The first exchange is the base exchange T to A base exchange (c.1799T>A), and the second is the addition of three base sets (c.1795 1796insCTA or c.1796 1797insTAC) that code for threonine between positions 598 and 599.^26^, ^27^ None of the clinical restrictions and the measurable relationships were observed (age, territory, and sex). The BRAF mixtures exhibited a strong association with the pediatric age group and the cerebellum in KIAA1549.^9^ Morphologically, the PA appeared more infiltrative, but due to the limited scope of our clinical follow‐up, we could not determine its detrimental prognostic significance. The most prominent tumor‐related alterations in the v‐RAF B1 (BRAF) gene for the murine sarcoma viral oncogene.^42^

#### 3.2.2 Neurofibromatosis type 1 (NF1) mutations

NF1 is a predominant problem that inclines victims to different types of neoplasia that influenced people, 15%–20% create astrocytoma, particularly CA (CA), which are generous and named I by the World Health Organization. NF1‐related PAs (NF1‐PAs) rarely act as aggressive tumors.^33^, ^43^, ^44^ It is believed that a combination of acquired physical and germline NF1 tumor silencer quality modifications causes CA to develop in neurofibromatosis type 1 (NF1) acquired malignancy inclination condition. However, cerebellar tumors produced by genetically engineered mice (GEM), in which biallelic physical (glial progenitor cell) Nf1 inactivation is paired with monoallelic germline Nf1 gene inactivation, do not entirely capture the neuropathological features of the human condition. The tumor inclination condition known as NF1 is associated with a variety of sensory system tumors as well as low‐grade gliomas (LGG) in the pediatric population. It is also associated with various clinical manifestations, such as bistro scafé‐au‐lait spots, intertriginous freckling, Lisch nodules, neurofibromas, optic pathway gliomas, and specific hard injuries. NF1 is triggered by a mutation that affects the neurofibromin encoder gene, a large protein involved within the MAPK and mTOR pathways through RAS‐RAF signaling cascades.^45^

The deficiency of 1 NF1 allele was recognized in 11 of 12 (92%) instructive NF1‐related pilocytic astrocytoma. On the contrary, only 1 of 24 educational (4%) irregular pilocytic astrocytoma showed allelic misfortune in the NF1 district. Among the 11 NF1‐related tumors with NF1 misfortune, 5 had likewise lost alleles on 17p.^46^ A 17q isodisomy is due to a single crossover between the NF1 and centromere genes. Due to the loss of the NF1 gene and deletions ranging in size from 80 kb to 8 Mb within 17q, LOH accounted for 38% of dogs with LOH (*N* = 49).^45^ In chromosome region 17q11.2, the NF1 gene produces a protein with tumor suppressor action. The loss of heterozygosity (LOH) for NF1 has been found in several neurofibromas and NF1 cancers.^47^

#### 3.2.3 Mutations in the Ras/ERK/MAPK pathway gene

Although BRAF fusion genes are the genetic anomalies that most frequently disrupt the Ras/ERK/MAPK pathway in sporadic PA, other mutations can also cause this pathway to activate.^48^ MAPK is activated in PAs through two distinct pathways. First, tandem duplication at 3p25 was found, strikingly similar to the typical BRAF fusion, which caused an oncogenic in‐frame fusion between SRGAP3 and RAF1. The Raf1 kinase domain is involved in the merger, which exhibits higher kinase activity than the wild type.^49^ In which the auto‐inhibiting RAF1 domain is replaced by the beginning of the SRGAP3 gene, the SRGAP3‐RAF1 fusion gene is established. Unlike KIAA1549—BRAF, SRGAP3—RAF1 does not have a transmembrane domain code, but contains the Fes/CIP4 (cell division control 42 protein‐interactive protein 4) homology domain.^36^, ^37^ In one pilocytic astrocytoma, the genes PTPN11, NRAS, KRAS, and HRAS were activated and somatic G12A KRAS mutations were found in Pas.^50^

### 3.3 Grade‐II; Diffuse astrocytoma/Low grade astrocytoma (LGA)

Diffuse astrcytoma, considered a slow‐growing brain tumor, is hypothesized to develop from astrocytes, develop in young adults, and have a chance to develop into more dangerous tumors over time.^51^, ^52^, ^53^, ^54^ The average survival time for infected patients is 5–8 years and they make up around 20% of all primary brain tumors.^55^, ^56^, ^57^ Common symptoms include headache, seizures, blurred vision, and speech problems for people with low‐grade astrocytoma.^58^, ^59^ Diffuse Astrocytoma can be further classified according to genetic diversity, such as the IDH1 or IDH2 mutation. However, diffuse astrocytoma can be treated depending on the type and size.

### 3.4 Molecular genetics and signaling mechanism behind low‐grade astrocytes

Several molecular genetic alterations and a signal account for low‐grade astrcytoma, and the three most indicative mutations are described in the following.

#### 3.4.1 Isocitrate dehydrogenase (IDH) mutation

IDH mutations and the loss of chromosomes 1p and 19q (referred to as 1p/19q codeletion) are the two characteristics that distinguish oligodendrogliomas, also known as diffuse gliomas, from other types of gliomas.^60^ The isocitrate dehydrogenase (IDH) mutation continues to play a significant role in the emergence of several gliomas, according to WHO.^61^ About 80% of grade II‐III gliomas and subsequent GBMs undergo the IDH mutation.^62^, ^63^, ^64^, ^65^ It appears that the IDH1 mutation in gliomas affects amino acid residue 132, and the major portion (more than 85%) of the mutation comprises a heterozygous change from arginine to histidine (R132H).^66^ This amino acid residue is located at the enzyme's active site, which is essential for isocitrate binding.^67^ The mutation at position 132 stops the protein's regular catalytic activity by inhibiting the protein's capacity to bind isocitrate. Consequently, the concentrations of some crucial cofactors, like ‐KG and NADPH, are reduced, yet the role of IDH2 in diffuse astrocytoma is unclear.^68^

#### 3.4.2 TP53 mutation

The TP53 mutation plays a dominant role in forming brain tumors.^69^, ^70^, ^71^, ^72^ This particular mutation has oncogenic characteristics, making it preferable for cell invasion, proliferation, metastasis, and immortalization. The tumor suppressor gene p53 disrupts the normal cell cycle by halting the cell in the late phase of G1. The mutation can also encourage apoptosis in cells with irreparable DNA damage.^73^, ^74^

#### 3.4.3 MGMT mutation

Controlling DNA damage from alkylating chemicals requires the O6‐methylguanine‐DNA methyltransferase (MGMT).^75^ The MGMT gene, which codes for O6‐alkylguanine‐DNA‐alkyltransferase, is found in the chromosomal band 10q26. The O6 position of guanine, a crucial target of alkylating and methylating chemicals, is where methyl and chloroethyl groups are removed by the repair enzyme known as AGT. Because it causes accumulation of DNA mutations and chromosomal instability, MGMT repair capacity deficiency contributes to the genesis and development of human malignancies.^76^, ^77^, ^78^ In diffuse astrocytoma, the mutation of the TP53 tumor suppressor gene mutation and the MGMT gene are prevalent.^79^

### 3.5 Anaplastic astrocytoma (AA)

Anaplastic astrocytoma, also known as grade III high‐grade astrocytoma, develops from star‐shaped glial cells called astrocytes.^80^ It evolves from the progression of low‐grade precursors or by de novo synthesis, accounting for 1% to 2% of all primary brain tumors.^81^ It induces from the cerebral hemisphere of the brain, but may occur in the central nervous system. The relevant cause of anaplastic astrocytoma is still unknown, but genetic and immunological deformities, stress, dietary habits, environmental factors like exposure to UV light, chemicals, ionizing radiations, a genetic disorder, for instance, NF1, tuberous sclerosis, and Li‐Fraumeni syndrome, hereditary predeposition may play a pivotal role in causing anaplastic astrocytoma according to the researcher's prediction. It is more prevalent in adults (30 to 50 years of age) than in children (5 to 9 years of age). Signs and symptoms of the tumor varied depending on its exact location and size. Seizures, eye problems, vomiting, speech changes, and changes in voice and mental state are some typical symptoms.^82^, ^83^

Anaplastic astrocytoma (AA) is divided into subgroups based on IDH mutation and 1p /10q co‐deletion; IDH mutated tumor and co‐deletion of 1p/10q with a better prognosis and wild type with the worst prognosis. Surgery is the main form of treatment for AA, followed by radiation and concurrent chemotherapy with temozolomide.^84^

### 3.6 Molecular genetics and signaling mechanism behind multiforme glioblastoma

#### 3.6.1 Mutation Tp53/ATRX in AA

**FIGURE 1:**
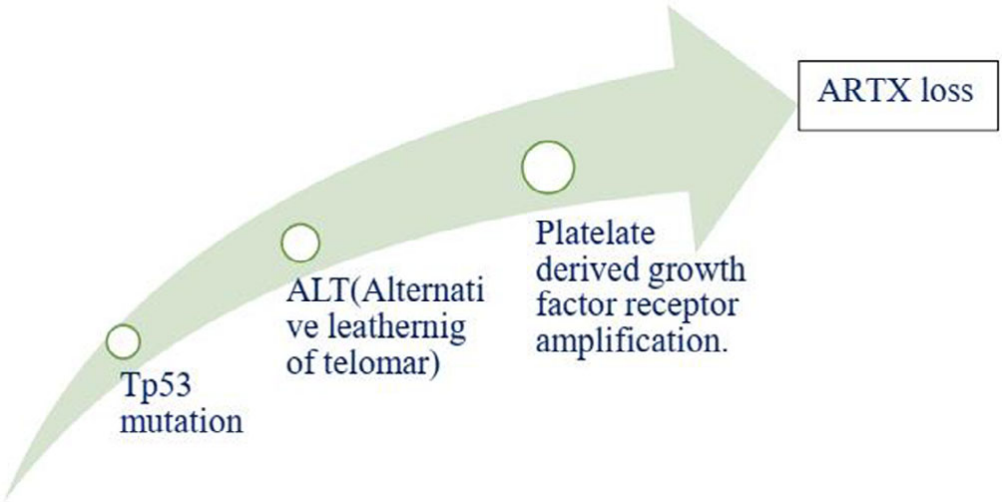
The above figure depicts the peak reason of ARTX loss, which is a considerable fact caused by TP53 mutation, ALT and PDGF receptor amplification, respectively.

Anaplastic gliomas that are more accurately classified by ATRX deletion defines a subgroup of IDH mutant astrocytic tumors with a longer useful life.^85^ To generate the ALT (−) and O6‐methylguanine‐DNA methyltransferase (MGMT)) phenotype, ATRX plays a crucial role.^86^ ATRX and the ALT phenotype were substantially linked. Although AOA with ATRX loss had a similar clinical outcome similar to AA, AOA with a co‐deletion of 1p/19q had a clinical course to AO. Mutation in TP53 53% and loss of ARTX 45% are the most common deregulation in AA glioblastoma.^87^ ATRX was co‐related with isocitrate dehydrogenase of single biomarkers (IDH), loss of heterozygosity [1p/19q co‐deletion], and O6‐methyl guanine DNA methyl transferase [MGMT].^88^ IDH immunohistochemistry is used for the detection of ATRX gene mutation. Diffuse astrocytoma is closely related to oligodendrogliomas WHO grade oligodendrogliomas (II & III).^89^, ^90^ The overall effect of the TP53 mutation is summarized in Figure 1. Since loss of ATRX is a distinguishing feature of astrocytic tumors, ATRX helps to better defining the symptomatic and structurally mixed group of AOA. ATRX deletion also identifies a subpopulation of astrocytic cancers with a good prognosis.

### 3.7 Multiform

Glioblastoma accounts for 50% of all gliomas and 20% of brain tumors and is one of the most prevalent and deadly primary intracranial neoplasms.^91^ Despite sophisticated treatment surgery followed by radiation therapy and concurrent chemotherapy with temozolomide, it remains untreatable, resulting in the patient's median survival (approximately 1 year).^92^ In 2007, WHO classified GBM as grade 4 high‐grade astrocytoma due to their aggressive nature, leading to a poor prognosis.^93^ It is mainly predominant in the elderly population; malesmen are more susceptible than females and hardly found in pediatrics. GBMs first evolve in the cerebral hemisphere, mainly in the frontal and temporal lobes–supratentorial regions. The cause is unknown; however, it affects people with genetic illnesses such as Turcot syndrome, neurofibromatosis Type 1, and Li‐Fraumeni syndrome. The therapeutic goals for glioblastoma multiforme are recognition and targeting of molecular pathology for proliferation and overexpression.^94^ In 1940 German neuropathologist Hans Joachim Sherer in Antwerp distinguished between primary and secondary glioblastoma from a biological and clinical perspective. In 2016, the WHO classified GBM in the following ways.^95^

• Glioblastoma, wild type refers to primary/de novo glioblastoma that accounts for 90% of the cases occurring predominantly in the older population (mean age over 55 years). Genetically manifests loss of heterozygosity 10 q (70%), EGFR amplification and overexpression (36%), PTEN mutation (25%).^96^
• Glioblastoma, the IDH mutant refers to secondary glioblastoma that occurs from lower grade diffuse glioblastoma or anaplastic astrocytoma that accounts for 10% of GBM evolving in the younger population (median age 45 years). It displays the Tp53 mutation (65%), the PTEN mutation (10%), and LOH 10q (63%). The LOH 10q mutation is the most common disorder among both gliomas. Tp53 (65%) is a primary genetic mutation that causes secondary glioblastoma. Malignancy is caused by the dysregulation of numerous signaling pathways, including the p53 system, loss of heterozygosity, the retinoblastoma pathway, and overexpression of the epidermal growth factor receptor, which is involved in growth, proliferation, survival, and death.^96^

### 3.8 Molecular genetics and signaling mechanism behind multiforme glioblastoma

#### 3.8.1 EGFR/AKT/PTEN/mTOR

EGFR is a central signaling cascade for primary glioblastoma (36%) and is hardly found in secondary glioblastoma involved in controlling cell proliferation, survival, apoptosis, and overexpression of EGFR. EGFR (HER1 or c‐ErbB1) is a transmembrane protein of the tyrosine kinase subfamily 1 receptor tyrosine kinase which comprises three main categories; extracellular ligand‐binding domain, the transmembrane domain, and an intracellular three‐cytoplasmic domain.^97^, ^98^

EGFR triggers when a specific ligand epidermal growth factor binds to the extracellular domain via homo and heterodimerization, which leads to the autophosphorylation of the the C‐terminal of tyrosine kinase domain due to dimerization of the cell surface culminating in the initiation of downstream signaling cascades such as PI3K, mTOR, and AKT pathways in activation of epidermal growth factor receptor. Genetic disruption, such as EGFR overexpression, loss of PTEN expression loss, and activation of the PI3K/Akt/mTOR pathways, leads to a poor prognosis.^99^

Phosphoinositide 3‐kinase, also called phosphatidylinositol 3‐kinase, is a crucial signaling pathway involved in cellular responses such as growth, metabolism, apoptosis, vesicular trafficking, and protein synthesis.^100^ A regulatory p85 subunit and a p110 catalytic subunit make up PI3K. Activated RTKs bind to p85 after binding a specific ligand to the receptor, activating the next catalytic subunit, p110. PIP3, a second messenger, is produced when activated phosphatidylinositol 3,4‐bisphosphate (PIP2) phosphorylates p110. PI3K antagonist PTEN when the reaction is reversed. Gleichzeitig, PIP3 triggers AKT and phosphorylates Akt (serine/ threonine kinase), leading to activation of mTORC1 for protein synthesis as well as cell growth. On top of this signaling pathway, the PTEN mutation overactivated the PI3K/Akt/mTOR pathway that contributes to GBMS.^101^ Amplification and overexpression of wild‐type EGFR VIII mutants produce numerous proteins that cause cancer.^102^

#### 3.8.2 RAS/MAPK signaling cascade in GBM

The G protein, related to the RAS family, is essential for controlling carcinogenesis, signal transduction, apoptosis, cell growth, proliferation, and differentiation. The RAS protein binds to GTP or GDP, which activates RAS and also regulates other essential pathways.^103^ Activated RAS activates RAF kinase, which controls downstream signaling pathways—the MAPK pathway. Dysregulation of the RAS/MAPK pathway leads to abnormal outgrowth and proliferation, evolving cellular responses such as apoptosis and metastasis.^104^

#### 3.8.3 Tp53/MDM2/P14ARF signaling cascade in GBM

Tp53; is a guardian of the gene responsible for the genomic stability of cell cycle control, DNA repair, apoptosis, and senescence. The Tp53 mutation is more common in secondary glioblastoma 65% of primary glioblastoma 28%.^105^ The alteration of the p53 pathway occurs through varying mechanisms. The P53 mutation in secondary glioblastoma multiforme is located at 248 and 253 codons, while primary glioblastoma multiforme is spread widely. These modifications demonstrate that the genomic instability of the GBM tumor microenvironment causes the p53 mutation in primary glioblastoma multiforme to develop as a secondary mutation. Additionally, they have different expression profiles and promoter methylation patterns at the RNA and protein levels.^106^ The MDM2 protein is encoded by the mouse double minute 2 gene, acting as a negative regulator of the p53 protein. mdm2 ligates with p53 protein, which ligates through E3 ubiquitin ligase and the remaining unliquidated p53, are degraded by proteasomes that maintain the stability of p53 signaling pathways.^107^ About 75% of patients with glioblastoma have P14 ARF abnormalities, including homozygous deletion and promoter methylation.^108^

**FIGURE 2:**
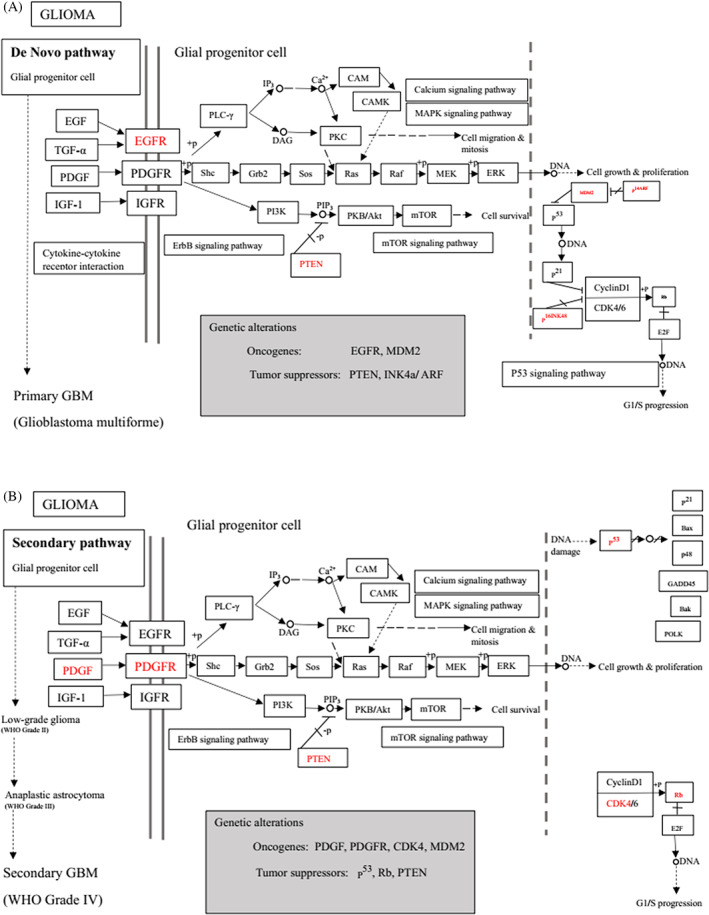
Figure shows the ultimate genetic alteration in normal glial cells where PTEN, ARF, and P53 conduct a crucial role as tumor suppressors genes. This figure is being generated from the KEGG pathway an online‐based tool, map no: map05214.^53^, ^54^, ^55^, ^56^, ^57^, ^58^, ^59^, ^60^, ^61^

Tp53 is activated when the DNA lesions occur; it causes transcription of genes‐p21 (waf1/Cip1). When MDM2 adheres to both mutant and wild‐type tp53 proteins, wild‐type tp53 activates transcription. The MDM2 genes are then transcribed as a result of Tp53.^109^ The p14 ARF gene product binds to mdm2 and inhibits mdm2 mediated p53 deregulation and transactivated silencing of mdm2. Henceforth, Tp53 is controlled by the expression of p14ARF and inversely correlates with the tp53 function.^110^ Due to reduced normal cell function, the deregulation of the tp53, MDM2 or p14ARF genes results in tumorigenesis.^111^ A summary of the major signals is presented in Figure 2 and Table 1.

**TABLE 1:** Some common mutations and signals related with astrocytoma development.

Histological class	Tumor types		Mutations			Major associated signals	References
			IDH1/2	1p/19q loss	Others		
Astrocytoma 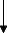 Oligoastrocytoma 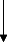 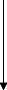 Oligodendroglioma 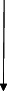	Low‐grade glioma (I‐II)	Diffuse astrocytoma	mut	Codel	TRET mut	Growth factor receptor a (PDGFRA), MET, CDK4, MDM2, and MDM4; mutations of phosphatidylinositol‐3‐OH kinase (PI3K); mutations and deletions of TP53, CDNK2A/ARF, CDKN2A/p16, RB1, and NF1	121, 122, 123, 124, 125
Anaplastic astrocytoma 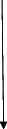 Anaplastic Oligoastrocytoma Anaplastic Oligodendroglioma	High grade glioma (III)		mut	wt	ARTX mut, P53 mut	Over expression of PDGF, PDGFR and EGFR, PI3K/Akt/mTOR pathways, loss of PTEN, RAS/MAPK signaling cascade and TP53, MDM2 or p14ARF	121
			wt	wt	p53 mut, PTEN mut, PIK3, EGFR amplification, CDKN2A/B deletion, CDK4, BRAF, ATRX mut		
Glioblastoma	High grade glioma (IV)		mut	wt	P53 mut		
			wt	wt	PTEN mut, EGFR amplification		

## 4 DIAGNOSIS

The patient's reports found a huge lack of exact elevated symptoms. As a result, the early detection of astrocytoma remains obscure. Some common neurological symptoms such as positional headache, visual abnormalities, and focal neurologic abnormalities such as numbness or weakness, nausea, and vomiting are generally seen in patients. In the diagnosis of astrocytoma, as well as tumor progression, brain biopsy is the only reliable method to measure the actual tumor progression, but it has a huge risk. In contrast, circulating biomarkers are also an excellent method to measure tumor progression though it is too costly. However, it is often done to determine the true progression of the tumor. MicroRNAs (Mi‐RNA) are the most widely used biomarkers of astrocytoma.^112^, ^113^

Imaging tests are used to help distinguish the tumor, comprehend the size of the tumor, and discover what cerebrum could be influenced. Magnetic resonance imaging (MRI) is the fundamental imaging procedure typically used to evaluate astrocytoma.^114^ The images made by MRI can give more data on the sort of tumor and the likely spread of infection. MRI is also done after a medical procedure to check whether any tumor remains. Computed tomography (CT) utilizes X‐beams to make cross‐sectional pictures of the organs and tissues inside the body. The machine takes numerous photos to make an extremely itemized picture. This can allow small tumors to be seen. A biopsy is typically used to analyze astrocytoma. A small piece of tissue is taken out during the medical procedure in a biopsy. A pathologist looks at the tissue test under a magnifying instrument to recognize the particular kind and grade.^27^ Imaging tests produce point‐by‐point pictures of fragile tissues, organs, and bones of the body. In youth sickness, these photos show the region of a tumor. The physicians called radiologists to explore the photos and give bare essential portrayals. Their reports help masters with diagnosing and treating youth threats. During treatment, the image shows the clinical gathering of how threatening development responds to treatment. After treatment, patients undergo imaging tests as part of ensuing visits to check for changes.^115^ After testing the cerebrospinal fluid (CSF), it assists the specialist in making a decision and makes him cautious about the tumor region (brain or spinal cord area), and will help the patient with valuable treatment if it is curable or not.

## 5 POSSIBLE THERAPEUTICS FOR ASTROCYTOMA

The anatomic region of a glioma impacts expectations and treatment decisions. Little is known about the risk of developing multicentric disease in patients with juvenile pilocytic astrocytoma (JPA) and even less about its prognosis.^116^ Before giving any treatment to the patient, it depends much more on the tumor's position, size, and area of predation. The clinical presentation of an astrocytoma is considerably influenced by its location in the brain than by its basic nature. In a review investigation of 73 patients who worked on cerebellar pilocytic astrocytoma, the consequences of treatment, the results, and the natural conduct of lingering tumors were broken down.^117^ Within a year after the medical procedure, CT or magnetic resonance scans in 69% of the cases clearly showed complete tumor excision.^117^ According to research, the best course of treatment for cerebellar pilocytic astrocytoma includes not only a clinical strategy aimed at complete tumor removal, careful tumor management to prevent the spread of tumor cells and the resulting metastases, and additional radiation therapy in specific chosen cases but also posttreatment outcomes dependent on direct postoperative neuroimaging, ideally MRI.^117^ Astrocytoma is delegated to second or high‐grade depending on what they look like under the magnifying lens. As a rule, astrocytoma treatment includes medical procedures to eliminate the tumor. Chemotherapy or radiation treatment is regularly utilized along with medical procedures to slaughter any outstanding disease cells. Different medicines can be utilized depending on the type of tumor. For the most part, second‐rate astrocytomas will generally be size before they become indicative in contrast to more forceful astrocytomas with higher evaluation. Lower‐grade tumors generally uproot the mind instead of devastating it, and are related to less cerebrum growth than threatening ones.^118^

According to the ESMO Guidelines, co‐deletions of 1p/19q, IDH mutations, and methylation of the MGMT promoter are frequently identified depending on the clinical and histological context. As surgery is the first therapeutic option for all malignant gliomas, the gold standard treatment for GBM is mixed modality therapy with TMZ and radiation.^119^ Exclusive TMZ chemotherapy may be recommended for older individuals with a methylation MGMT promoter, while (hypofractionated) radiation is the preferred therapy for people with an unmethylated gene promoter.^120^ On the contrary, there has not been any evidence supporting the use of adjuvant PCV chemotherapy (procarbazine, lomustine [CCNU], vincristine) in the treatment of anaplastic astrocytoma. In the case of anaplastic glioma, adjuvant chemotherapy alone and radiation alone when the tumor has progressed are equal to the conventional regimen of initial radiation therapy and salvage chemotherapy when the tumor has advanced.^20^, ^121^, ^122^ Recent research demonstrated that MGMT methylation, regardless of tumor grade, is a reliable indicator of chemotherapy efficacy in patients with wild‐type IDH.^122^

### 5.1 Signaling‐based treatments

**FIGURE 3:**
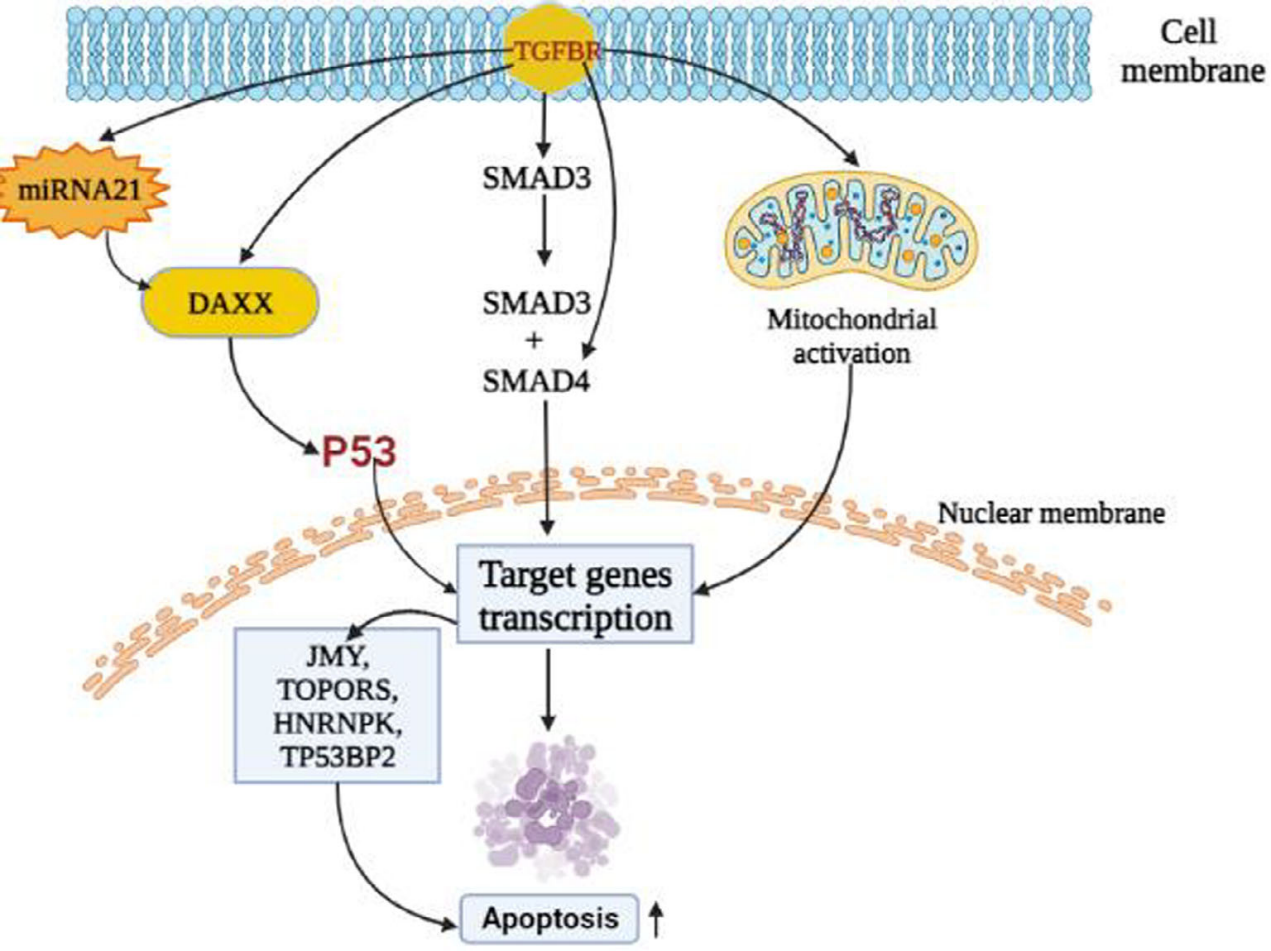
Signaling‐based treatment mechanism regarding astrocytoma. Basically these signaling pathways like TGFBR, miRNA, SMAD are interconnected to each other. Activation/inhibition of one singnal either promote or impede another signaling cascade, leading to halt cancer progression.

Some encouraging findings have been made from the accomplished research on miRNA in the management of glioma. MiRNAs are tiny noncoding, naturally occurring components of RNA comprised of 18–24 nucleotides that control gene expression at the post‐transcriptional level.^123^, ^124^ Numerous types of astrocytoma, such as pilocytic, diffuse, anaplastic, and multiform glioblastomas, have been associated with various miRNA expression patterns. When miRNA expression is downregulated, it displays tumor suppressor activity; when it is upregulated, it is oncogenic. All recent studies show that microRNA contributes to the determination of gene expression and identifies a potential therapeutic target for malignant gliomas.^125^, ^126^ In short, miRNA has been used as a biomarker over the years because of its variety of expression in different tissues. Countless miRNA targets have been identified in the case of astrocytoma, and their involvement in the differentiation, proliferation, and apoptotic processes of tumors has demonstrated their contribution to tumor growth.^127^, ^128^, ^129^ It is the most reliable formula for glioma diagnosis and therapeutic progress.^130^ Recent research on high‐grade gliomas: GBM based on patient serum revealed that tumor cells emit microvesicles containing miRNAs, among which we may mention miR‐15b, miR‐16, miR‐21, miR‐26a, miR‐27a, miR‐92, miR‐93, and miR‐320. miR‐21, −132, −134, −155, −210, and −409‐5p have excessive expression in GBM.^131^, ^132^ miRNAs are associated with important pathways such as EGFR, p53, AKT, TGF‐dependent, and nuclear NF‐kB.^133^ MiR‐21 is the most common microRNA associated with the most important signaling pathways. According to several studies in recent years, high levels of miR‐21 have been found in most cancers and glioblastoma tumor cells. Chan et al. investigated the assertion of miR‐21 in a particular form of GBM and glioma cells. They found that miR‐21 expression was five to 100 times higher in neoplastic brain tissues than in healthy ones.^134^, ^135^, ^136^ According to our study, they play a vital role in the formation of gliomas such as the AKS pathways, the TGF‐β signaling pathways, and the p53 pathways are directly associated with the miR‐21 microRNA. Up‐regulation of miR‐21 is mainly responsible for this.^135^, ^137^, ^138^, ^139^ Some studies show that, for p53 activation of p53 in the pathway, the true response has been seen by the appearance of miR‐21 and some transcripts.^140^, ^141^ The TGF‐pathway, specifically TGFBR2, TGFBR3, and DAXX, were putative targets of miR‐21. When the TGF‐ligand binds to TGFBR2 and TGFBR3, these receptors are activated, and by activating SMAD transcription or DAXX, respectively, they can limit growth or induce death. Tumor suppressing p53 homologous TP73L (TAp63), as well as the activating cofactors JMY, TOPORS, HNRPK, and TP53BP2, were expected targets for the p53 pathway^140^, ^142^, ^143^, ^144^, ^145^, ^146^ In the presence of antisense oligonucleotide, inhibition of miR‐21 consequences in the compression of EGFR pathways. It also causes glioma proliferation.^130^, ^147^ MiR‐221 and miR‐222 are also responsible for AKS and p53 pathways. Down‐regulation of miR‐221 or miR‐222‐mediated MGMT could polish off glioma cells to make them incapable of repairing genetic damage.^148^ MiR‐451 is important in increasing cell survival by activating the AMPK pathways. The downregulation of miR‐451 in glioma samples is also inversely correlated with grades of malignant glioma.^149^ In this study, miR‐15b, miR‐21, and miR‐221 were examined and were considered oncogenic potential, while miR‐124, miR‐128, miR‐137, and miR‐221 were considered tumor suppressors.^128^, ^150^, ^151^, ^152^, ^153^, ^154^ The general process by which the signal can regulate the growth of an astrocytoma is shown in Figure 3.

### 5.2 Available surgery and treatment

We mentioned above that there are four types of astrocytoma, so surgery and possible therapy will depend on these grades and aggressive conditions. So, depending on these grades, doctors can suggest available conventional treatments such as surgery, radiation therapy, chemotherapy, and so forth. A person treated for astrocytoma may also need physical and occupational therapy.

PA are the most prevalent type of grade I astrocytoma. These tumors have well‐defined edges. They also rarely become a higher‐grade astrocytoma. Therefore, pilocytic astrocytomas are often cured with surgery. However, a person carrying grade (I) or pilocytic astrocytoma does not need immediate surgery. In this case, after surgery, no further treatment is needed. If the tumor was not fully removed, doctors can think about additional available therapy systems and can suggest that patients receive radiation therapy or another targeted therapy.^155^

The best treatment for grade II diffuse astrocytoma is still unclear. A gathering of prosperity masters or surgeons can discuss the best treatment for patients. This gathering may include a neurosurgeon, radiation oncologist, clinical neurooncologist, and various pros. The authorities acknowledge that clinical methodology is still essential for assurance and therapy. The primary objective is to eliminate enough tissue for end‐ and condition monitoring.^3^ If assessment II astrocytoma returns, the clinical methodology may be another option. Therapy after the clinical system depends on the patient's treatment history. The patient may receive radiation, chemotherapy, or both. Radiation, chemotherapy, or both may be obtained if a clinical strategy is not another option.

If astrocytoma III or IV returns, treatment decisions depend on where the sickness is and its degree. The clinical system may be a decision followed by chemotherapy, radiation, or glioblastoma, using electric field therapy. When cautious therapy is not a decision, various options consolidate chemotherapy, the clinical technique for results, turning electric field treatment (glioblastoma), and consistent thought.^155^ Glioblastoma, the most notable basic psyche tumor in adults, is normally rapidly deadly. High‐grade gliomas (HGG) have a poor prognosis. At the time of study of glioblastoma, conservative excision to the extent possible is the current standard of care, followed by adjuvant radiation. The most effective neuro‐oncology test may be the one for redundant treatment with glioblastoma multiforme (rGBM) treatment.^156^

### 5.3 Experimental therapeutics

Along with advances in medical science, treatment has increased. Therefore, it becomes easier for physicians to provide the proper treatment depending on the characterization of the grade, location, and size.

After chemotherapy, monoclonal immunotherapy has become popular in this field. An attractive treatment option for glioblastoma is immunotherapy with safe checkpoint inhibitors such as ipilimumab, nivolumab, and pembrolizumab, which have significantly improved clinical outcomes in other advanced malignancies where traditional medicines have had limited success.^157^ Over the past 10 years, research into the genetics and epigenetics of GBM has shown abnormalities in cell signaling pathways, the tumor microenvironment, and neurotic angiogenesis. Numerous targeted anticancer drugs, including monoclonal antibodies and small atom kinase inhibitors, have been evaluated in clinical trials with recently studied intermittent GBM.^158^ Among the most dangerous and aggressive brain tumors with few treatment options is glioblastoma multiforme. Maximal meticulous resection, concurrent adjuvant chemoradiotherapy, and support therapy with temozolomide constitute the standard of care. With post‐surgical radiation alone, this methodology increases the evaluation with medium and 5‐year endurance. Additional prognostic and predictive indicators are essential, especially in light of the development of targeted therapies such as antibodies and tyrosine kinase inhibitors. These innovative and practical methods are being closely examined. The most positive data currently available relate to anti‐angiogenic medications, such as bevacizumab and cediranib. This study provides an overview of the potential role in managing glioblastoma multiforme.^159^ Chemotherapy utilizing temozolomide is currently the gold standard for treating GBM. The drug is often administered every day during radiation treatment and again for 6–12 cycles after radiation. Each cycle lasts 28 days, the first 5 days of which are spent in temozolomide, while the next 23 days are spent at rest. Although chemotherapy aims to control long‐term tumors, only around 20% of patients achieve this.^160^ The list of those tested clinically are summarized in Table 2.

**TABLE 2:** Drugs tested for astrocytoma are listed with outcomes.

Registration No.	Recruitment status	Name of the drug	Treatment	Research components	Main outcomes
NCT02104310	Active, not recruiting	Fluorine‐18‐L‐dihydroxyphenylalanine		Participants = 25 Age = 7 Years and older Sex = All	Able to distinguish tumors from normal brain tissue. Identification of the most aggressive regions of the tumor.
NCT03528642	Active, not recruiting	Telaglenastat Hydrochloride Temozolomide	Phase 1	Participants = 40 Age = 16 Years and older Sex = All	Halt the growth of tumor cells by blocking some of the enzymes needed for cell growth
NCT02796261	Active, not recruiting	Eflornithine Lomustine	Phase 3	Participants = 343 Age = 18 Years and older Sex = All	Comparison of the efficacy and safety of eflornithine in combination with lomustine
NCT03197506	Recruiting	Temozolomide	Phase 2	Participants = 52 Age = 18 Years and older Sex = All	Halt the growth of tumor cells, either by killing the cells, stopping them from dividing, or stopping them from spreading
NCT01553149	Active, not recruiting	Lenalidomide	Phase 2	Participants = 75 Age = up to 21 Years Sex = All	Stop the growth of tumor cells by blocking blood flow to the tumor.
NCT02414165	Terminated	Toca FC Lomustine Temozolomide	Phase 2 Phase 3	Participants = 403 Age = 18 Years to 75 Years Sex = All	Assess the safety and effectiveness of combined Toca 511 and oca FC, versus a standard of care single agent chemotherapy
NCT00892177	Completed	Dasatinib Bevacizumab	Phase 2	Participants = 144 Age = 18 Years and older Sex = All	Stop the growth of tumor cells by blocking some of the enzymes needed for cell growth and by varieties of way
NCT04910022	Recruiting	NMS‐03305293 + TMZ Lomustine	Phase 1 Phase 2	Participants = 125 Age = 18 Years and older Sex = All	Determination of the antitumor effects and Maximum Tolerated Dose (MTD) and the Recommended Phase 2 Dose (RP2D) of NMS‐03305293 in combination with temozolomide (TMZ)
NCT04047264	Recruiting	NA	NA	Participants = 52 Age = 18 Years and 89 Years Sex = All	Pharmacokinetics Evaluate the safety of the devices and the feasibility of their use to collect analytes with sizes up to 100 kDA.
NCT02444546	Completed	Sargramostim	Phase 1	Participants = 6 Age = 10 Years to 21 Years Sex = All	Enhance the production of blood cells and promote the tumor cell‐killing effects of wild‐type reovirus.
NCT04197934	Recruiting	EGFR/EGFRvIII Inhibitor WSD0922‐FU	Phase 1	Participants = 77 Age = 18 Years and older Sex = All	Determination of the maximum tolerated dose (MTD) and/or the recommended Phase 2 dose (RP2D) of WSD0922‐FU for the treatment of glioblastoma, anaplastic astrocytoma, or non‐small cell lung cancer.

### 5.4 New promising and alternative therapies

#### 5.4.1 Antiangiogenic therapy

High blood vessels and glioblastomas contain a protein called vascular endothelial growth factor (VEGF), which promotes the creation of new blood vessels (the process of angiogenesis). Drugs that block the formation of new blood vessels also encourage the absorption of existing blood vessels. In clinical trials, several antiangiogenic drugs have been tried, with encouraging preliminary results in both newly diagnosed and chronic HGG.^161^

#### 5.4.2 EGFR‐targeting antibody drug

EGFR is a desirable therapeutic target due to its critical role in the survival of malignant cells. Clinical trials are now being conducted for first‐line and recurrent GBM disease on Depatux‐m, which has demonstrated encouraging clinical efficacy in patients with GBM.^162^

#### 5.4.3 Natural products act as an advanced and alternative therapy

Although some of the available treatments for treating the brain that have been described before are ineffective and have a number of negative effects, the inability of synthetic medications to penetrate the blood–brain barrier (BBB), which is extremely impervious to foreign molecule entrance, hinders their ability to treat patients.

**FIGURE 4:**
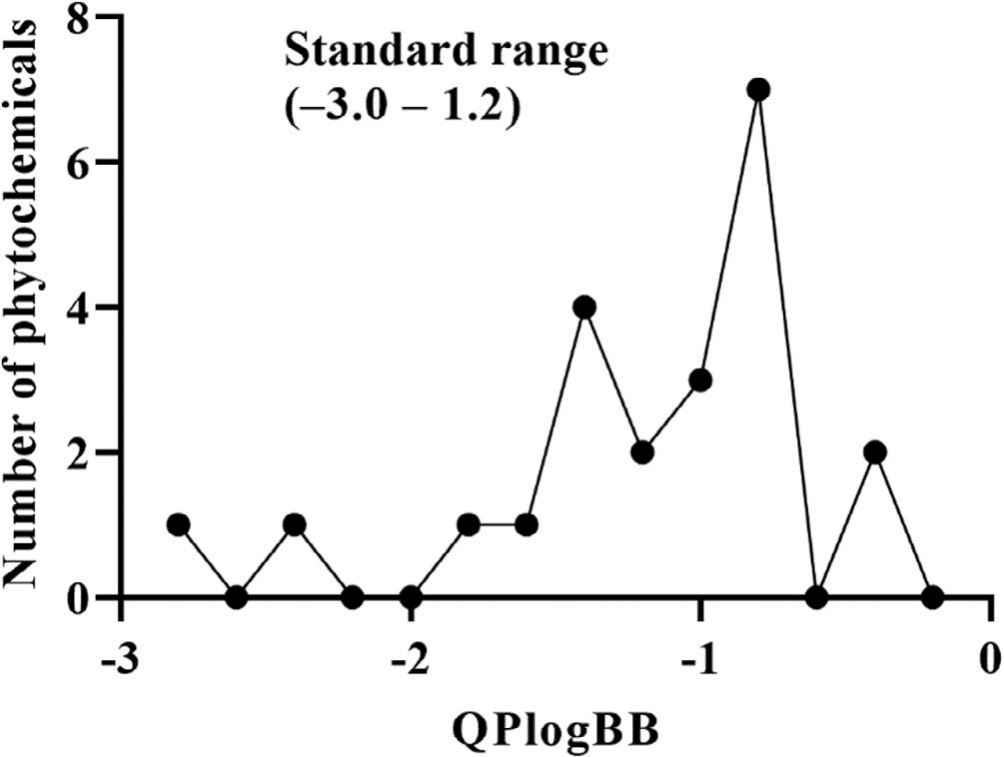
Predicted brain/blood partition coefficient. At the X axis, Q PlogBB indicates permeability of blood brain barrier and Y axis for phytochemicals count. This figure was generated by Schrodinger Release 2021–2: Maestro, ¨ Schrodinger, LLC, New York, NY, 2020–3 and Graph pad prism.

Numerous chemical substances found in natural goods can penetrate the blood–brain barrier and are believed to control the BBB microenvironment around brain tumors and aid in their treatment. As a result, we chose a few possible phytochemicals found in nature (Table 3) that can pass the blood–brain barrier (Figure 4). Through a variety of methods, such as transcellular diffusion, carrier‐mediated transcellular transport, or paracellular distribution that employs tight junctions between BBB endothelial cells, several natural substances can pass through the BBB.^163^

**TABLE 3:** Overview of phytochemicals activities in astrocytoma treatment and management.

Phytochemical name	Therapeutic dose	Study model (in vitro/in vivo)	Molecular mechanism	Molecular target	References
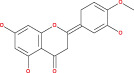 Hesperetin	100–800 μM	In vitro (U‐251, U‐87)	↓ Cell Viability ↑ Apoptosis	↑ Bax protein ↓ Bcl‐2 ↑ cell cycle Arrest G2/M phase ↓ Cyclin B1, CDK1 ↑ p21, p38 MAPK	164
	10–20 mg/kg	In vitro/In vivo (Wistar rat with c6)	↑ Apoptosis ↓ Cell proliferation	↑ Caspase‐9, ‐3 ↑ Claudin‐1, ZO‐1 expression ↑ Bax and Bcl2 ratio ↓ HIF‐1α, VEGF, VEGFR2	
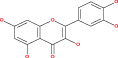 Quercetin	50 mg/kg	In vitro (U87MG, CHG‐5 cells)	↓ Migration ↓ Invasion	↓GSK‐3β/β‐catenin/ZEB1	180
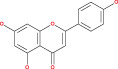 Apigenin	(0–50 μM)	In vitro (U87)	↓ Invasion, migration ↑apoptosis cell death.	↑ Cdk‐Cyclin mediated G2/M phase arrest and ↑ ROS‐mediated apoptosis ↑ Bax/Bcl‐2 ratio	181
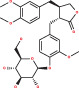 Arctiin	(0–50 μM)	In vitro (U87)	↓ Invasion, migration ↑apoptosis cell death.	↑ Cdk‐Cyclin mediated G2/M phase arrest and ↑ ROS‐mediated apoptosis ↑ Bax/Bcl‐2 ratio	181
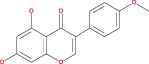 Biochanin A	0–100 mmol/L	In vitro (U251)	↑ Apoptosis ↓ Migration and invasion ↓ Cell proliferation	↑ Bax and ↓ expression of p‐Akt and p‐mTOR	182
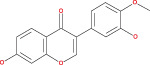 Calycosin	800 μM	In vitro (U251)	↓Cell proliferation and cell invasion ↑Cell apoptosis	↓Transforming growth factor beta (TGF‐β), N‐cadherin, Snail, Vimentin respectively ↓Mesenchymal properties and MMPs respectively	184
		U87 cells			
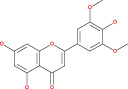 Chrysin	120 μM	In vitro (T98, U251, U87)	↓Cell proliferation ↓cell invasion ↓ migration	↓ Nrf2/ERK signaling pathway	185
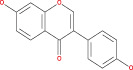 Daidzein	80 μM	In vitro (U87M, U251, U‐118MG, A‐172)	↓ Cell proliferation ↑G0/G1 arrest	↓ Bcl2 ↓ CD44/moesin/β‐catenin signaling pathway	186
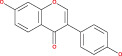 Formononetin	0 ~ 200 μM	In vitro (U87MG, U251MG, and T98G)	↓ Cell proliferation	↓ HDAC5 expression	187
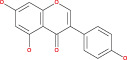 Genistein	10 μM	In vitro (M059K, U87 U251)	↓ HR repair pathways	↓ DNA‐PKcs phosphorylation ↓ NHEJ and delaying HR repair pathways	188
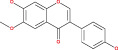 Glycitein	50 μM	In vitro (U87MG)	↓ DNA synthesis ↑ G2/M cell cycle arrest, apoptosis	↓ Protein levels of MT1‐MMP and uPAR ↓ MMP‐2 and MMP‐9	181
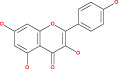 Kaempferol	120 μM	In vitro (U87 MG, U251)	↓Proliferation ↑ ROS generation ↑Apoptosis ↑DNA damage	↑ pro‐caspase3 ↑mRNA levels of IL‐1β and ASC ↓ cleavage levels of GSDME	189
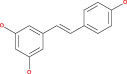 Resveratrol	100 μM	In vitro (U251 and LN428)	Remarkable growth arrest and extensive apoptosis	↑ intracellular ROS levels and attenuated SOD2 and catalase expression	175
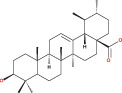 Ursolic acid	5–20 μM	In vitro (U251)	↓Proliferation ↑ Apoptosis ↓ Cell growth	↓ TGF‐b1/ miR‐21/PDCD4 Pathway ↑Activation of caspase‐3	176
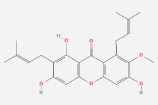 α‐mangostin	2 mg kg^−1^ day^−1^	In vitro and in vivo (GBM8401 and DBTRG‐05MG)	↓ Cell viability ↑ Inhibition of cell growth ↑ Autophagic cell death ↓ Apoptosis	↓ mTORC1, p70 ribosomal protein S6 kinase (p70S6 kinase) and 4E‐BP1 ↑ LKB/AMPK pathway	178
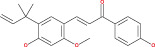 Licochalcone A	30 μM	In vitro (U87)	↑ Cell cycle arrest in the G0/G1 and G2/M phases ↓Cell growth	↓Cyclins and CDKs	177
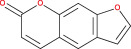 Psoralen	(30 μM)	In vitro (U87, U251)	↓Proliferation, migration, carcinogenic gene expression ↑ Apoptosis, cell cycle	↓ expression of PIK3CA, PIK3CB, PIK3CG, JAK2 gene and PI3K, JAK2 ↓ protein phosphate‐3‐kinase/protein kinase (PI3K/Akt)	187
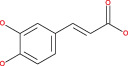 Caffeic acid	50 mM	In vitro (C6)	↓Cell growth ↓Cell cycle progression	↓Hyperphosphorylated pRb decreased ↓Cyclin‐dependent kinase ↑Inhibitors p21, p27, and p16	179
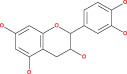 Catechin	20 μM	In vitro (U‐87)	↓ Invasion	↓ RhoA/ROK ↑RhoA and MT1‐MMP	180
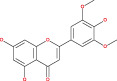 Chrysin	0, 30, 60, and 120 μM	In vitro (U87, T98, U251, U87)	↓Proliferation, migration, invasion	↓ Nrf2 (Nrf2/ARE pathway) ↓ Hemeoxygenase‐1 (HO‐1) and NAD(P)H quinine oxidoreductase‐1 ↓ ERK1/2	185
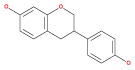 Equol	20 μM	In vitro (C6)	↑Apoptosis ↑Neuronal death	↓LPS ↑TLR4 activation, JNK phosphorylation ↑expression of Bax and cleaved caspase‐3 and ↓Bcl‐2 ↑Neuroinflammation	182
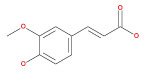 Ferulic Acid (FA)	36 μM	In vitro (U‐87 MG)	↑Apoptosis ↓Cytotoxicity	↓Bcl‐2, ERK1/2, c‐Myc ↑ PARP‐1 cleavage	183
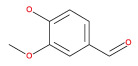 Vanillin	100 μM	In vitro (GL261)	↓Cell growth ↑Proliferation	↓TLR2 ↑MMP 9, MMP 14, IL 6, and iNOS expression	184
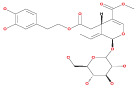 Oleuropein	0, 200, and 400 μM	In vitro (U251 and A172)	↑Apoptosis ↓ Invasion and migration ↑ Anti invasive effect ↓Cell growth and proliferation	↓Phosphorylation of AKT (p‐AKT) ↑Bax ↓Bcl‐2 ↓Matrix metalloproteinase‐2 (MMP‐2) and MMP‐9, phosphatidylinositol 3 kinase (PI3K), LY294002	186
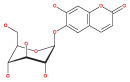 Esculin	360 μM	In vitro (U87)	↓ Adhesion and migration ↓ Cell viability ↓ Capillary tube formation	Effecting the attachment α2β1 and αvβ5 integrins	185

Hesperetin, the in vitro cell line U‐251, U‐87 at a dose ranging from 100 to 800 μM has the mechanism to decrease cell viability and in increasing the Bax protein, lowering Bcl‐2 and cyclin B1, CDK1 while increasing G2/M phase cell cycle arrest and p21, p38 MAPK.^164^ Quercetin reduces cell migration and invasion by decreasing the GSK‐3β/β‐catenin/ZEB1 pathway in vitro, respectively, in U87MG and CHG‐5 cell lines at a dose of 50 mg/kg.^165^ Apigenin and arctiin at a dose of 0–50 μM reduce cell migration, invasion leading to apoptosis and cell death increasing CDK‐Cyclin mediated G2/M phase cell cycle of the G2/M phase mediated by CDK‐Cyclin, generating ROS and the Bax/Bcl‐2 ratio in the invitro technique inin the glioma U87 cell line.^166^ Biochanin A at a dose of 0–100 mmol/L in vitro glioma cell line U251 causes cell apoptosis while reducing cell migration, invasion, and proliferation, respectively. Biochanin A has been observed to suppress p‐Akt and p‐mTOR expression, whereas it up‐regulates the Bax protein.^167^ Calycosin decreases cell proliferation and invasion simultaneously, inducing apoptotic cell activity in a dose‐dependent manner in cell lines U251 and U87.^168^, ^169^ Chrysin down‐regulates Nrf2/ERK signaling pathway by decreasing migration, invasion, and cell proliferation based on the glioma cell lines T98, U251, and U87 invitro at a periodic dose of 0–120 μM respectively.^170^ Daidzein induces the arrest in the cell cycle, while it lowers the cells proliferation in glioma cell U87M, U251, U‐118MG, and A‐172 at a dose of 0–20, 40, and 80 μM, respectively, by downregulating the expression of Bcl‐2 and CD44/moesin /β‐catenin signaling pathway in vitro manner.^171^ Formononetin shows little cytotoxicity and reduces the cell proliferation in the glioma cell lines U87MG, U251MG, and T98G by lowering HDAC5 expression in vitro at a dose of 0–200 μM.^172^ Genistein, in the glioma cell line U87MG at a dose of 10 μM, increases the arrest of the G2/M cell cycle and cellular apoptosis by downregulating the protein levels of MT1‐MMP and uPAR, as well as MMP‐2 and MMP‐9.^173^ Kaempferol inhibits proliferation and accelerates ROS generation to increase apoptosis by triggering DNA damage by increasing the levels of IL‐1β and ASC as well as decreasing the cleavage level of GSDME cleavage at a dose of 120 μM.^174^ At a dose of 100 M, resveratrol induces significant apoptosis and cell growth arrest in the in vitro cell lines U251 and LN428. It also lowers the expression of SOD2 and catalase.^175^ At a dose of 5–20 μM, ursolic acid inhibits the TGF‐b1/miR21/PDCD4 pathway and increases caspase‐3 activation, inducing cell proliferation and apoptosis while inhibiting cell growth in the invitro cell line U251.^176^ In the invitro cell line, ursolic acid inhibits the TGF‐b1/miR21/PDCD4 pathway, increases caspase‐3 activation, and induces cell proliferation and apoptosis while suppressing cell growth. U251.^177^ At a dose of 2 mg kg^−1^ day^−1^, alpha‐mangostin (α‐mangostin) reduces cell viability and promotes apoptosis, cessation of cell growth, and autophagic cell death by lowering Mtorc1, P70S6 kinase (p70S6 kinase) and 4E‐BP1. It also partially boosts the LKB/AMPK pathway.^178^ By reducing cell growth along cell cycle progression in invitro cell c6 glioma, caffeine at a dose of 50 mM reduces hyperphosphorylation of pRb, and cyclin‐dependent kinase increases inhibitors of p21, p27, and p16.^179^ Calycosin inhibits TGF‐N‐cadherin, Snail, Vimentin, MMP‐2 and MMP‐9 and inhibits glioma cell invasion and migration in vitro using the glial cell lines U87 and U251 in a dose‐dependent manner.^168^ In vitro, catechin reduces the RhoA/ROK ratio, increases RhoA and MT1‐MMP, and decreases cellular invasion in the cell line U‐87 in a dose‐dependent manner between 100 and 200 mM.^180^ Glycerin inhibits DNA synthesis, increases arrest of the G2/M cell cycle, and promotes apoptosis in glioma cell line while decreasing the protein levels of MT1‐MMP and uPAR, as well as MMP‐2 and MMP‐9.^181^ Equol prevents LPS activity, induces TLR4 activation and JNK phosphorylation, upregulates the expression of Bax and cleaved caspase‐3, downregulates the expression of Bcl‐2, and, when administered at a specific dose of 20 μM, increases apoptosis and neuronal death in vitro glioma cell line C6. This causes neuroinflammation.^182^ At a dose of 36 M, ferulic acid (FA) causes cellular apoptosis and decreases the expression of Bcl‐2, ERK1/2 and c‐Myc while increasing the expression of PARP‐1 cleavage in the glioma cell line U‐87MG.^183^ By decreasing cell growth and enhancing cellular proliferation, vanillin at a dose of 100 μM can decrease TLR2 expression and increase the expression of MMP‐9, MMP‐14, IL‐6, and iNOS expression in the glioma cell line GL261 at a dose dependent.^184^ Esculin minimizes adhesion, migration, cell survival, and capillary tube formation that affect the attachment of integrins 2 and 5 when used at a dose of 360 μM in the invitro glioma cell U87.^185^ Oleuropein was reported to be anticarcinogenic in vitro in cell lines U251 and A172 at doses of 0, 200, and 400 μM doses. By reducing the phosphorylation of AKT (p‐AKT), Bcl‐2, matrix metalloproteinase‐2 (MMP‐2), MMP‐9, phosphatidylinositol 3 kinase (PI3K) and LY294002, it exhibits an increase in apoptosis, anti‐invasive action and decreases in invasion, migration, cell growth and cell proliferation, respectively.^186^ By suppressing the expression of the PIK3CA, PIK3CB, PIK3CG, JAK2 gene, PI3K, JAK2, and protein phosphate‐3‐kinase/protein kinase (PI3K/Akt) cascades, also mentioned in Wu et al., 2022, Psoralen can reduce cell proliferation, migration and carcinogenic gene expression, induce apoptosis, and potentially trigger cell cycle arrest.^187^ An overview of phytochemical activities in the treatment and management is summarized in Table 3.

## 6 LIMITATIONS AND FUTURE DIRECTION FOR THE TREATMENT OF ASTROCYTOMA

Knowledge about signaling‐based astrocytoma is limited. Before establishing a standard drug, a large and clinical‐based study is needed to unravel the main oncogenic signal. In this regard, the determination of protein–protein interaction by network pharmacology can be effective. In terms of treatment, surgery continues to be the preferred treatment method among available treatments, although it is not without drawbacks.^188^ Other options are radiation therapy, experimental therapies like adjuvant therapy, and therapy, which also have some limitations. Therefore, there are several challenges in treating patients with astrocytoma. Only certain small and highly lipophilic chemicals can enter the brain, making conventional therapy approaches difficult. Most cytotoxic drugs used in cancer therapy (in traditional formulation) typically have lower BBB penetration capacities and are linked to nonspecific distribution to nearly all other essential organs/tissues, compromising treatment outcomes with unavoidable significant healthy tissue harm. Nanoparticles (NPs) are at the forefront of potential future treatments for GBM since current therapies for the disease are still therapeutic rather than curative. Nanobased therapies for cancer treatment have progressed since the FDA approved the first generation of nanomedicines, Doxil® (1995) and Abraxane (2005). The efficacy of delivering therapeutic compounds to the brain with a new generation of NPS has been studied to circumvent the restrictive characteristics. More laboratory‐based research on signal biology and astrocytoma is needed to make a final decision.^189^

## 7 CONCLUSION

To conclude, astrocytoma basic biology has become clearer owing to current research in the field of cancer, particularly in brain tumors, and there are now chances of its quick translation into clinical benefits for patients. This indicates that information regarding signaling cascade‐based death of cancer patients is still lacking. However, our efforts did not go in vain or without success, and we can conclude that four different forms of astrocytoma are directly related to any oncogenic signaling cascades. The MAPK cascade, the leader of Ras‐Raf‐ERK signaling pathways, is a common signaling mechanism in astrocytoma, leading to activation of PDGFR, EGFR, AKT, PTEN, and PTEN. BRAF mutations, such as KIAA1549: BRAF and BRAF V600E have been linked to the progression of astrocytoma, according to our investigation. Like other tumors, the development of astrocytoma is significantly influenced by dysregulations in some tumor suppressor genes, including the Tp53/ATRX mutation and the MGMT mutant. Conventional treatments are not so effective due to some limitations, and we focused on some natural compounds that have BBB permeability. The accumulation of data suggests that many biological proteins, including cell cycle regulatory (cyclin B1, CDK1), apoptosis (Bax protein, Bcl‐2, caspase3, caspase‐9, ‐3 and ROS), tumor suppressor protein (p16, p21, p27, p38, and p53), signaling (p‐Akt, p‐mTOR, AMPK, PI3K/Akt, metastatic factor (MMP‐2 and MMP‐9) and growth factor receptor (VEGF, VEGFR2) are simultaneously targeted by some phytochemicals, which limit tumor formation. Although alteration of the BRAF and MAPK pathway causes brain tumors, targeting therapies are now recognized as potential novel treatment approaches. There are currently several preliminary phase I/II clinical trials that are ongoing that are testing small molecule kinase inhibitors targeting MAPK or related pathways, including MEK inhibitors (ClinicalTrials.gov: NCT01386450, NCT01089101), RAF/multiple tyrosine kinase inhibitors such as Sorafenib (ClinicalTrials.gov: NCT01338857), and mTOR inhibitors in patients with and without NF1 (ClinicalTrials.gov: NCT01158651, NCT00782626). The holistic outcome of these early clinical studies on the biology of astrocytomas and the development of new treatment options offer hope for improving outcomes in patients with this type of brain cancer.

## AUTHOR CONTRIBUTIONS

**Chowdhury Lutfun Nahar Metu:** Conceptualization (equal); writing – original draft (equal). **Sunita Kumari Sutihar:** Writing – original draft (equal). **Md Sohel:** Conceptualization (lead); visualization (equal); writing – original draft (equal); writing – review and editing (equal). **Fatematuz Zohora:** Writing – original draft (equal). **Akayed Hasan:** Writing – original draft (equal). **Md. Thandu Miah:** Writing – original draft (equal). **Tanu Rani Kar:** Writing – original draft (equal). **Md Arju Hossain:** Data curation (equal). **Md Habibur Rahman:** Supervision (lead); visualization (equal); writing – original draft (equal); writing – review and editing (equal).

## CONFLICT OF INTEREST STATEMENT

The authors have stated explicitly that there are no conflicts of interest in connection with this article.

## ETHICS STATEMENT

Not applicable.

## Data Availability

Data sharing is not applicable to this article as no new data were created or analyzed in this study.
